# Isolation and Characterization of Bacteriocin-Producing *Lacticaseibacillus rhamnosus* XN2 from Yak Yoghurt and Its Bacteriocin

**DOI:** 10.3390/molecules27072066

**Published:** 2022-03-23

**Authors:** Yonghua Wei, Jinze Wang, Zhe Liu, Jinjin Pei, Charles Brennan, A.M. Abd El-Aty

**Affiliations:** 1 College of Food Science, Shanxi Normal University, Taiyuan 030031, China; weiyonghua1983@126.com; 2 Qinba State Key Laboratory of Biological Resources and Ecological Environment, Qinling-Bashan Mountains Bioresources Comprehensive Development C.I.C., Shaanxi Province Key Laboratory of Bio-Resources, College of Bioscience and Bioengineering, Shaanxi University of Technology, Hanzhong 723001, China; 15029368245@163.com (J.W.); l1115711913@163.com (Z.L.); 3 College of Food Science and Engineering, Royal Melbourne Institute of Technology, Melbourne 3046, Australia; charles.brennan@rmit.edu.au; 4 Department of Pharmacology, Faculty of Veterinary Medicine, Cairo University, Giza 12211, Egypt; abdelaty44@hotmail.com; 5 Department of Medical Pharmacology, Faculty of Medicine, Atatürk University, Erzurum 25240, Turkey

**Keywords:** *Lacticaseibacillus rhamnosus*, yak yoghurt, antibacterial activity, purification, bacteriocin

## Abstract

Lactic acid bacteria (LAB) produce antimicrobial substances that could potentially inhibit the growth of pathogenic and food spoilage microorganisms. *Lacticaseibacillus rhamnosus* XN2, isolated from yak yoghurt, demonstrated antibacterial activity against *Bacillus subtilis*, *B. cereus*, *Micrococcus luteus*, *Brochothrix thermosphacta*, *Clostridium butyricum*, *S. aureus*, *Listeria innocua CICC 10416*, *L. monocytogenes*, and *Escherichia coli*. The antibacterial activity was estimated to be 3200 AU/mL after 30 h cultivation. Time-kill kinetics curve showed that the semi-purified cell-free supernatants (CFS) of strain XN2 possessed bactericidal activity. Flow cytometry analysis indicated disruption of the sensitive bacteria membrane by semi-purified CFS, which ultimately caused cell death. Interestingly, sub-lethal concentrations of semi-purified CFS were observed to reduce the production of α-haemolysin and biofilm formation. We further investigated the changes in the transcriptional level of *luxS* gene, which encodes signal molecule synthase (Al-2) induced by semi-purified CFS from strain XN2. In conclusion, *L. rhamnosus* XN2 and its bacteriocin showed antagonistic activity at both cellular and quorum sensing (QS) levels. Finally, bacteriocin was further purified by reversed-phase high-performance liquid chromatography (RP-HPLC), named bacteriocin XN2. The amino acid sequence was Met-Lue-Lys-Lys-Phe-Ser-Thr-Ala-Tyr-Val.

## 1. Introduction

A yak (*Bos mutus*) is a long-haired bovid commonly farmed in the Himalayan region. It is distributed in an area above the altitude of 3000 m in the Tibet Plateau in China [1]. Homemade and naturally fermented yak milk yoghurt is one of the favorite foods for the Tibetan people. The yoghurt is prepared by naturally fermenting yak milk in a custom-made, specially treated tung-made big jar for at least 7–10 days at ambient temperatures around 15 °C to produce acidity, alcohol, and flavor to the desired level [2]. A characteristic common feature of this yak yoghurt is the presence of alcohol in addition to lactic acid [3]. These yak milk yoghurts in the Qinghai-Tibet Plateau area may be considered a good reservoir for lactic acid bacteria. Ding et al., (2011) investigated the microorganisms among the yoghurts from Gansu province in China [1]. Among the isolates, 164 isolates (51.41% of the total) were classified under *Lactobacilli*, and 155 (48.59%) belonged to *cocci*. All the isolates were classified into six genera (*Lactobacillus*, *Lactococcus*, *Leuconostoc*, *Streptococcus*, *Enterococcus*, and *Weissella*) and 21 species. Luo et al. (2017) indicated that the average counts of lactic acid bacteria of the naturally fermented yak milk were higher than that of yoghurt and suggested new strains of lactic acid bacteria and yeasts may be isolated from the yak yoghurts, as well as for new functional products [2].

Many researchers are screening and isolating bacteriocin-producing lactic acid bacteria from naturally fermented yoghurt due to concerns of potential damage to health by artificial chemical preservatives [4,5]. Bacteriocins from lactic acid bacteria are peptides secreted by some lactic acid bacteria with antimicrobial activity against other microorganisms, including food spoilage and pathogens. They are generally considered safe [6]. Todorov et al. isolated bacteriocins ST461BZ and ST462BZ produced by *L. rhamnosus* isolated from boza [7]. Srinivasan et al. reported that *L. rhamnosus* L34 isolated from the feces of Thai Breastfed infants could produce bacteriocins [8]. However, there is no report on screening bacteriocins producing lactic acid bacteria from naturally fermented yak yoghurts. This study aimed to screen and isolate bacteriocin-producinger lactic acid bacteria (LAB) from the naturally fermented yak yoghurt in the Qinghai-Tibet plateau in China.

Most of the research regarding the antibacterial mechanism action of bacteriocins has focused on their mechanism of action against sensitive bacteria cells. For example, they damage the integrity of the cell membrane and bind to the DNA [4,5]. However, some researchers denoted that bacteriocins may also inhibit biofilm formation and the production of secondary metabolites. For instance, *Lactiplantibacillus paraplantarum*, isolated from cheese, produces bacteriocin FT259, potentially influencing *Listeria monocytogenes* biofilm formation [9]. Chopra et al. [10] investigated a new bacteriocin with the potential to prevent biofilm formation. This study aimed to characterize the antibacterial activity and the mode of action of the bacteriocin-producing strain isolated from naturally fermented yak yoghurt and its bacteriocin. As biofilm formation may be regulated by the quorum sensing (QS) system [11,12], we also investigated whether bacteriocins were also able to regulate the QS system of sensitive bacteria in this study. 

## 2. Results and Discussion

### 2.1. Screening of Bacteriocin Production by LABs Isolated from Yak Yoghurt

Among the isolates from Qinghai yak yoghourt, strain XN2 could inhibit the growth of both gram-positive and gram-negative bacteria (the diameter of the inhibition zone was 15.1 ± 1.2 mm for *S. aureus* CICC10384, and 7.2 ± 0.5 mm for *E. coli* CICC10302). After eliminating the antibacterial activity caused by organic acids or H_2_O_2_, strain XN2 was selected as the bacteriocin producer. Based on carbohydrate utilization profile using API 50 CHL kits and 16S rRNA sequence analyses, the strain XN2 was identified as *L. rhamnosus*, referred to as *L. rhamnosus* XN2.

Naturally fermented yak yoghurts from different locations may contain different microorganisms. For instance, Ding et al. (2011) reported that *Lactobacillushelveticus*, *Leuconostoc mesenteroides* subsp. *mesenteroides*, *Streptococcus thermophilus*, *Lactobacillus casei*, and *Lactococcus lactis subsp. lactis* were the predominant populations in the yak milk products from Gansu Province in China [1]. This study isolated the functional strain XN2 from yak yoghurts in Qinghai province. The use of *L. rhamnosus* in food has been well documented in probiotics and starter culture strain, and it is generally accepted as a safe and probiotic lactic acid bacteria [7,8,13].

The activity spectrum is listed in Table 1. The semi-purified CFS of stain XN2 deployed the inhibitory activities towards both gram-positive (*Bacillus subtilis*, *B. cereus*, *B. megaterium*, *Micrococcus luteus*, *S. aureus*, *Listeria innocua*, and *L. monocytogenes*) and gram-negative bacteria (*E. coli*) (Table 1).

It is worth noting that the semi-purified CFS of strain XN2 could inhibit gram-negative (*E. coli*), while most bacteriocins from LAB belonged to Class I and Class II, only inhibiting gram-positive bacteria [14,15]. As well as bacteriocin from strain XN2, some bacteriocins were also found to have the ability to interact with intracellular enzyme systems and nucleic acid, besides damaging the integrity of the bacterial cell membrane. The determination and characterization of these bacteriocins with broad antibacterial spectra require the attention of more and more researchers [16,17]. One of the bottlenecks of applying bacteriocins in the food industry or medical clinics is that the antibacterial spectra of bacteriocins is usually narrow. The ability of the strain XN2 and its bacteriocin, which was active against both gram-positive and negative, might improve its potential.

### 2.2. Bacteriocin Characterization of XN2 Strain

#### 2.2.1. Growth and Bacteriocin Production Curve

**Figure 1:**
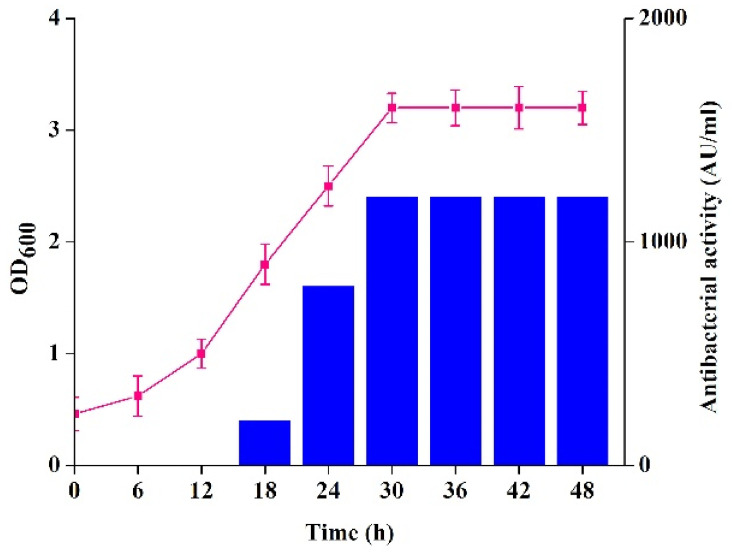
Production of bacteriocin by strain XN2. Square: growth of strain XN2; column: antibacterial activity of the CFS of strain XN2. The data are shown as mean ± standard deviation. The critical limit is 5%. *p* < 0.05, the differences are significant.

Bacteriocin production began after 8 h, and the stable high level was recorded after 30 h of growth in MRS broth (Figure 1). Similar results were also reported for other LAB bacteriocin-producing strains, such as *Companilactobacillus crustorum* MN047 [17], *Lactobacillus plantarum* ST16Pa [18], and *Pediococcus acidilactici* HW01 [19]. Bacteriocin might be the secondary metabolites of lactic acid bacteria.

#### 2.2.2. Bacteriocin Stability

Treatment with proteinase resulted in inactivation, whereas treatment with catalase, α-amylase, and lipase did not affect the antibacterial activity (Table 1). Similar results were observed for bacteriocin RC20975 produced by *L. rhamnosus* RC20975 [13] and bacteriocin BacC1 produced by *Enterococcus faecium* C1 [20]. The antibacterial activity was stable under heat treatment and over a pH range from 2–8 (Table 1). However, bacteriocin RC20975, similar to Sonorensin, enterocin E50-52, and pediocin PA-1 [10,13,21] were quickly inactivated under alkaline conditions

### 2.3. Kill Kinetics Curve of Semi-Purified CFS of Strain XN2 against S. aureus

**Figure 2:**
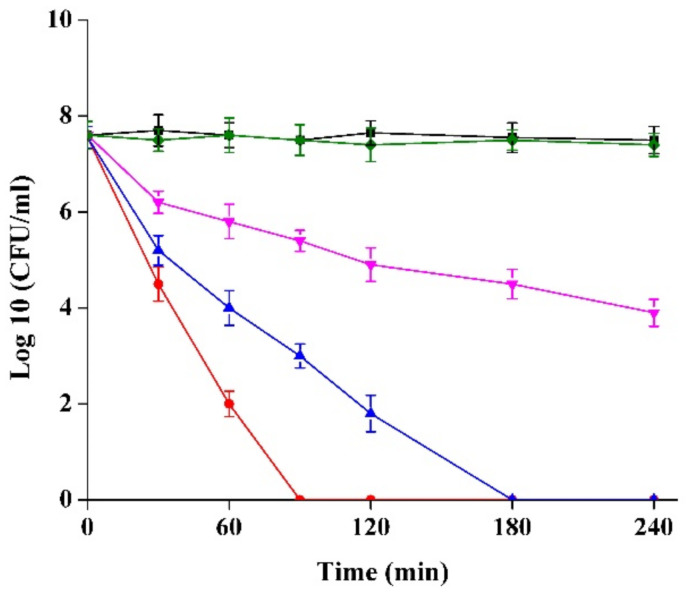
*S. aureus* in the PBS treated with 0 μg/mL (◆), 50 μg/mL (■), 100 μg/mL (▼), 200 μg/mL (▲) and 400 μg/mL (●) of the semi-purified CFS of strain XN2. The data are shown as mean ± standard deviation. The critical limit is 5%. *p* < 0.05, the differences are significant.

Treatment with 50 μg/mL semi-purified CFS of strain XN2 did not affect the population of *S. aureus*. However, treatment with 100 μg/mL semi-purified CFS of strain XN2 inhibited the growth of *S. aureus*. On the other hand, the population of *S. aureus* was drastically decreased within 1 h of treatment with 200 or 400 μg/mL semi-purified CFS (Figure 2).

The bactericidal activity against *S. aureus* implied that the inhibitory effect of the semi-purified CFS was concentration dependent. Similar action was reported for Enterocin FH99 isolated from *Enterococcus faecium* FH99 (active against *L. monocytogenes)* by Kaur et al. (2013) [22], bacteriocin RC20975 produced by *L. rhamnosus* RC20975 (active against *Alicyclobacillus. Spp.)* by Yue et al. (2013) [13], and Plantaricin GZ1-27 produced by *L. plantarum* GZ1-27 (against *Bacillus cereus)* by Du et al. (2018) [16].

### 2.4. Effect of Semi-Purified CFS of Strain XN2 on the Integrity of S. aureus

**Figure 3:**
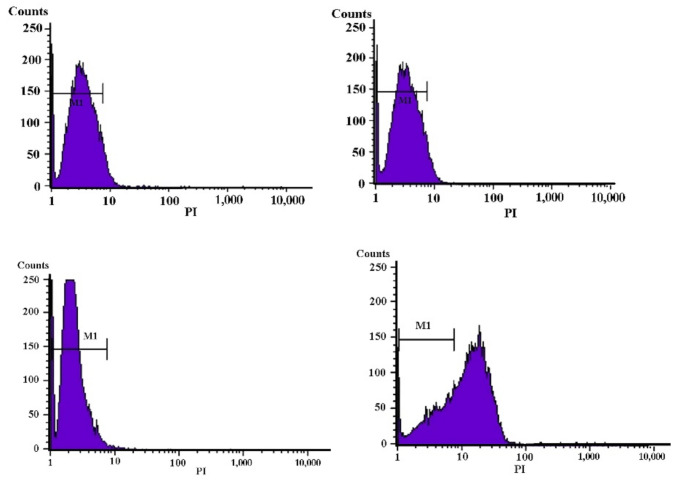
Effects of the semi-purified CFS of strain XN2 on the membrane integrity of *S. aureus*, tested by fluorescent staining and flow cytometry. Left-up: *S. aureus* cells treated with 2 MIC of the semi-purified CFS of strain XN2 for 0 min; right-up: for 30 min; left-down: for 60 min; and right-down: for 240 min.

Living cells cannot allow the passage of *propidium iodide* (PI) across the intact cell membrane. However, increase in permeability would increase the concentration and fluorescence intensity of PI within the cells. Herein, an extension of the incubation time led to a significant increase in the amount of PI passed through the cells; the finding was proved by the cell membrane rupture (Figure 3). These results indicate that the semi-purified CFS of strain XN2 can increase the cell permeability, ultimately resulting in cell death.

The great diversity of chemical structures of bacteriocins from different lactic acid bacteria determines the diversity of their functions and modes of action [17,23]. However, researchers believe that the alteration of cell membrane permeability is the principal mechanism of action of bacteriocins isolated from LAB [24,25]. Similar mode of action was also seen in Bacteriocin RC20975 from *Lacticaseibacillus rhamnosus* CICC20975, bacteriocin BacC1 from *Enterococcus faecium* C1, and bacteriocin SLG10 from *Lactobacillus plantarum* SLG10 [13,20].

### 2.5. Effect of Semi-Purified CFS of Strain XN2 on α-Haemolysin Secreted by S. aureus

**Figure 4:**
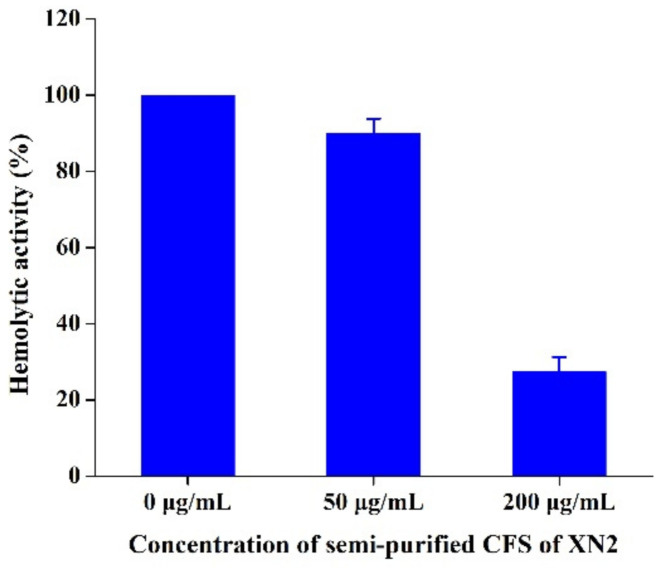
Effects of semi-purified CFS of strain XN2 on the secretion of α-haemolysin by *S. aureus*. The data are shown as mean ± standard deviation. The critical limit is 5%. *p* < 0.05, the differences are significant.

Compared with the control, the hemolytic activity of *S. aureus* CICC 10384 decreased to 90.05% and 27.34%, following treatment with 1/2 MIC and 2 MIC semi-purified CFS of strain XN2, respectively (Figure 4).

These results could justify the ability of the semi-purified CFS of strain XN2 to inhibit the secretion of α-haemolysin in a dose-dependent manner, even at the sub-MIC. α-haemolysin is one of the major virulence factors produced by *S. aureus* [26]. Semi-purified CFS of strain XN2 reduced the secretion of α-haemolysin, which meant that bacteriocin from stain XN2 had good potential characteristics to control *S. aureus* in the future.

### 2.6. Effect of Semi-Purified CFS of Strain XN2 on the Biofilm Formation of S. aureus

**Figure 5:**
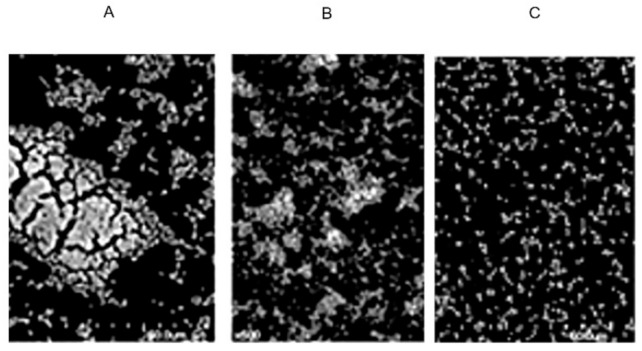
Effect of semi-purified CFS of strain XN2 on the biofilm formation of *S. aureus*; (**A**): control (*S. aureus* cells treated with no semi-purified CFS); (**B**): with 1/2 MIC semi-purified CFS; and (**C**): with 2 MIC semi-purified CFS.

In the control group, *S. aureus* (Figure 5A) was able to form a thin biofilm. It should be noted that samples treated with 1/2 MIC semi-purified CFS of strain XN2 would have fewer bacteria attached to the carrier surface (Figure 5B). The inhibition percentage of biofilm formation of *S. aureus* by the same concentration of semi-purified CFS of strain XN2 was 45.8 ± 3.1. The results were consistent. For the sample treated with 2 MIC semi-purified CFS of strain XN2, it could be seen that there were fewer cells in the biofilms, the biofilm was looser (Figure 5C), and the inhibition precent of biofilm formation was 90.3 ± 5.9. This finding suggested that bacteriocin XN2 could inhibit the formation of biofilm.

It is well known that only a tiny proportion of bacteria are present in the form of free-floating plankton [9]. The formation of biofilm communities is one of the most critical strategies for microbial survival under a particular ecological niche [11]. As strain XN2 and its bacteriocin could inhibit biofilm formation of food pathogens, such as *S. aureus*, they might be used in the food industry. The biofilm formation and α-haemolysin secretion may be regulated by the QS system [12]. Thus, it can be assumed that strain XN2 and its bacteriocin can also regulate the QS system of sensitive bacteria. Therefore, we further investigated the effect of semi-purified CFS of strain XN2 on the QS system of *S. aureus.*

### 2.7. Effect of Semi-Purified CFS of Strain XN2 on the QS System of S. aureus

**Figure 6:**
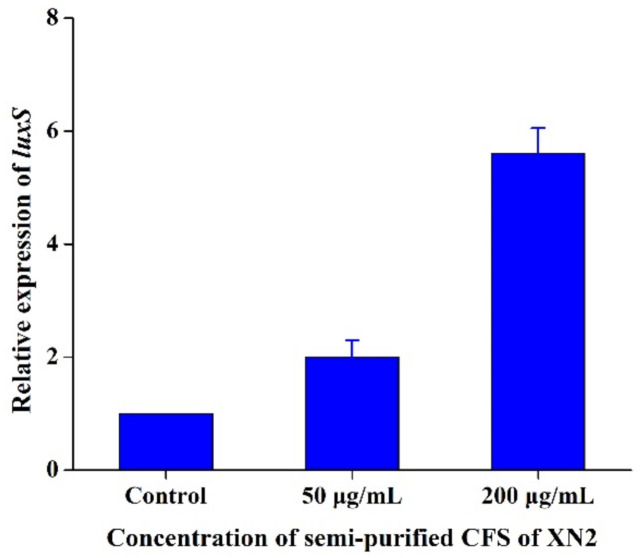
Effect of semi-purified CFS of strain XN2 on the relative expression of *luxS* of *S. aureus*. The data are shown as mean ± standard deviation. The critical limit is 5%. *p* < 0.05, the differences are significant.

With 1/2 MIC or 2 MIC semi-purified CFS of strain XN2, the gene expression of *luxS* was increased 2 and 5.6 fold (Figure 6). The result proved that strain XN2 or its bacteriocin could affect the QS system of *S. aureus* at a molecular level.

It was reported that the expression of gene *luxS* is one of the vital targets for QS inhibitors. Therefore, the effect of bacteriocin on the *luxS* gene expression was tested to determine the encoding of the protein LuxS, the enzyme for the synthesis of Al-2, and the signal molecule of the QS system. It was generally accepted that the formation of biofilm and production of some secondary compounds, for instance, α-haemolysin, were regulated by the QS system. When the bacterial population was big enough, inferred by the amount of signal molecules, the processing of the biofilm formation and production of secondary compounds, like α-haemolysin, starts. The results in this study were complementary with the findings that semi-purified CFS of strain XN2 can affect the secretion of α-haemolysin and the formation of biofilm aforementioned above. Most of the previous research has focused on bacteriocin’s inhibition/killing mechanism on sensitive bacteria via increasing the membrane permeability and eventually cell death [14,18]. There are few reports investigating the effect of bacteriocin on the QS system of sensitive bacteria [9]. In the present study, a bacteriocin from XN2 has dual activities, damaging the integrity of *S. aureus* cells and affecting its QS system.

### 2.8. Purification of Bacteriocin

After (NH_4_)_2_SO_4_ precipitation, the specific activity of bacteriocin improved to 262.44 IU/mg. After passing through the Sephadex G-50 column, fractions 33 to 37 showed antibacterial activity. A single sharp peak in the RP-HPLC spectrum at retention time of 16.58 min showed the antibacterial activity.

The three-step method used in this study for isolation and purification of bacteriocin XN2 was frequently used and was utilized for successful purification of diverse bacteriocins from culture supernatants, such as bacteriocin RC20975 [13], Plantaricin GZ127 [16] and bacteriocin BacC1 [20]. The industrial production of nisin, the most well-known bacteriocin, was also obtained by the standard three steps. The standard purification method would make this bacteriocin easier to obtain.

N-sequencing indicated that the amino acid sequence of the bacteriocin was Met-Leu-Lys-Lys-Phe-Ser-Thr-Ala-Try-Val (MLKKFSTAYV). Similarity analysis revealed none in the database (http://web.expasy.org/blast/, accessed on 5 February 2019). Therefore, this bacteriocin was designated as a novel peptide, bacteriocin XN2. Bacteriocin XN2 falls into the category of Class IId bacteriocins because it does not contain lanthionine or YGNGVXC (characteristics of Class IIa bacteriocins).

The mode of action of small-sized bacteriocins has recently attracted much research attention. Due to the small size, bacteriocins might not cause “pore formation” on sensitive bacteria. They might inhibit cell wall/membrane synthesis through binding with precursors of cell wall/membrane synthesis, destroying the integrity of the wall/membrane, and interacting with enzyme system or DNA in cells [27].

## 3. Materials and Methods

### 3.1. Isolation and Identification of Bacteriocin-Producing Stain

Traditional Natural fermented Qinghai yak yoghurts (a total of 40 bottles from 20 different local markets (GaoYuanZhiBao Co.Ltd, Xining, China)) were used to isolate bacteriocin-producing LAB. One loop of the yoghurt was inoculated into 20 mL MRS medium and incubated anaerobically at 37 °C for 24 h. Then, one loop was streaked onto a De Man, Rogosa, Sharpe (MRS) agar (Oxoid, Basingstoke, UK) plate. Gram-positive, catalase-negative, and oxidase-negative bacterial strains were chosen as LAB [27]. Afterwards, cell-free culture supernatants (CFS) from LAB were produced by centrifugation (Avanti J-E, Beckman, CA, USA) at 5000× *g* for 15 min at 4 °C, followed by 0.22 μm micro-filtration (Millipore, MA, USA), according to Yue et al. [13]. The pH of the CFS was adjusted to pH 6.5 to eliminate the antibacterial effect of organic acid. Catalase (Solarbiio, Beijing, China) was added to eliminate the effect of peroxide, as described by Yue et al. [13]. Furthermore, the agar well diffusion method was utilized for the antibacterial activity screening of CFS. Gram-positive, *S. aureus* CICC10384, and Gram-negative bacteria, *E. coli* CICC 10302, were used as indicator bacteria [28]. The indicator strains were pre-cultured at 30 °C to mid-exponential with anaerobic culture. The well with 10 mM phosphate buffer (pH 6.5) was used as a negative control. The well with 2 MIC Ampicillin (Solarbiio, Beijing, China) in buffer was considered as a positive control. The strain in which CFS showed a clear inhibition zone with both indicator strains was chosen and isolated. Primary strain identification was performed using a commercial kit (API 50 CHL, BioMerieux, Montalieu Versie, France), which recognizes the carbohydrate fermentation pattern of bacteria [29]. Finally, the bacterial strain genotype was identified by 16S rRNA gene sequence analysis using the same 27F (5′-AGTTTGATCMTGGCTCAG-3′) and 1492R primers (5′-GGTTACCTTGTTACGACTT-3′) as reported earlier [7]. Sequence similarity searches were compared with NCBI (www.ncbi.nlm.nlh.gov, accessed on 9 January 2018) database.

### 3.2. Bacteriocins Production by Strain XN2

A 12 h old culture of strain XN2 was inoculated (5%, *v*/*v*) into MRS broth and incubated at 30 °C. Cell growth, antibacterial activity, and pH value of the culture were tested at 6 h intervals [13]. The growth of strain XN2 was evaluated by testing the optical density at 600 nm (OD_600_). Antibacterial activity was tested with *S. aureus* CICC10384 as the sensitive strain. The pH values of the culture were monitored with a pH meter (Lei-ci SJ-5, INESA Scientific instrument Co., Ltd., Shanghai, China). 

### 3.3. Semi-Purification of CFS of Strain XN2

The CFS of strain XN2 was semi-purified using ammonium sulfate ((NH_4_)_2_SO_4_) precipitation and size-exclusion chromatography (SEC) [13]. After 30 h cultivation, the CFS of strain XN2 was recovered by centrifugation at 10,000× *g* for 30 min at 4 °C. Afterwards, CFS was concentrated to 1/5th of the initial volume using a rotavapor (RV-8V, IKA, Staufen, Germany). Subsequently, (NH_4_)_2_SO_4_ was slowly added up to the concentration of 60% (*v*/*v*). After stirring overnight at 4 °C, the precipitate was collected and resuspended in 20 mM Na_2_HPO_4_-citric acid buffer (pH 3.6). The biologically active solution was added onto the Sephadex G-50 column (80 × 2.0 cm, Sigma, Santa Clara, CA, USA) and eluted by Na_2_HPO_4_-citric acid buffer at a flow rate of 0.5 mL/min. The highest active fraction was collected and freeze-dried. The Bradford protein assay method was used [30] to quantify protein in a fraction. The antibacterial activity was assessed by the agar well diffusion method using *S. aureus* CICC10384 as an indicator strain [7].

### 3.4. Antimicrobial Spectrum and Stability Testing

The antibacterial spectrum was determined using indicator strains, including *Bacillus subtilis* CICC 10034, *B. cereus* CICC 2155, *Micrococcus luteus* CICC 10209, *Brochothrix thermosphacta* CICC 10509, *Clostridium butyricum* CICC 10350, *Staphylococcus aureus* CICC 10384, *S. aureus* CICC 10201, *Listeria innocua* CICC 10416, *L. monocytogenes* CICC 21529, *Escherichia coli* CICC 10302, *E. coli* CGMCC 3373, *E. coli* CICC 10300, *Pseudomonas aeruginosa* CICC 21636, *Enterobacter cloacae* CICC 21539, *Salmonella paratyphi* β CICC 10437, *Aspergillus niger* CICC 2124, *Candida albicans* CICC 1965, *Saccharomyces cerevisiae* CICC 1002 All the strains are purchsed from China Center of Industrial Culture Collection(CICC). To determine the influence of pH or enzymes on antibacterial activity, the pH of the samples was adjusted between 2 and 10 with 1 M HCl and 1 M NaOH solutions, or the samples were treated with different enzymes (Lipase, α-Amylase, Proteinase K, Papain, α-Chymotrypsin, Trypsin, Pepsin, and Catalase) (Solarbiio, Beijing, China), with the final concentrations of 1.0 mg/mL, as described by Du et al. [18]. The effect of temperature on antibacterial activity was tested, as shown in Table 1. The antibacterial activity was tested as described above, with S. aureus CICC 10384 as indicator strain [7]; samples without any treatments were used as controls.

### 3.5. Time-Killing Kinetics

Overnight-cultured *S. aureus* CICC 10384 was washed with and suspended in PBS (10 mM, pH 7.0), adjusted to the OD_600_ of 0.5, and treated with 50, 100, 200, and 400 μg/mL of semi-purified CFS of strain XN2. At fixed time points, the plate count method determined viable cells [13]. 

### 3.6. Flow Cytometry (FCM) Analysis

The mid-logarithmic phase of *S. aureus* CICC10384 at a concentration of 10^4^ CFU/ mL was co-cultured with 2 MIC semi-purified CFS of strain XN2 at 37 °C for 240 min. Cells were then labeled with propidium iodide (PI) and subjected to flow cytometry analysis (Becton Dickinson, NJ, USA), as described by Pei et al. (2018) [27,28,31]. Untreated *S. aureus* CICC 10384 cells were used as a control. 

### 3.7. α-Haemolysin Secretion

*S. aureus* CICC 10384 was inoculated in LB broth containing semi-purified CFS of strain XN2 at 1/2 MIC and 2 MIC (control without semi-purified CFS of strain XN2) and was cultured at 30 °C for 24 h. Following filtration with 0.22 um filter membrane, the *α*-haemolysin content was tested according to Zhou et al. (2017) [26]. Fresh fibrin removed from rabbit blood was washed with calcium chloride buffer three times. A mixture of 875 μL calcium chloride buffer, 100 μL sample supernatant, and 25 μL red blood cells was incubated for 30 min at 37 °C, centrifuged at 5500× *g* 1 min, then tested for the D_543_ value.

### 3.8. Biofilm Formation

The mid-logarithmic phase culture of *S. aureus* CICC 10384 was diluted to approximately 10^7^ CFU/mL. Each 6-well cell culture plate hole was filled with 1 mL of the suspension. The cell culture plate was pre-positioned with a sterile cover glass (22 mm × 22 mm). Plates were cultured at 30 °C after adding 0 (control), 1/2 MIC, or 2 MIC of semi-purified CFS of strain XN2. After 25 h of cultivation (visible and mature biofilm can be seen in control samples), the cover slides were taken out from each hole and washed with distilled water three times. After fixation with 2.5% glutaraldehyde and washing with PBS, the samples were then dehydrated with ethanol (100% repeated thrice, 90, 80, 70, and 50%) for 15 min each. The gold powder was used to cover the sample surface under vacuum conditions. Samples were observed under a scanning electron microscope. 

The percent of biofilm inhibition was assessed by the crystal violet staining method according to the protocol adopted by Saporito et al. (2018) [32]. Briefly, after stained by crystal violet (0.1% *w*/*v* in water) for 10 min at room temperature (a washing step with PBS removed the excess dye) and re-dissolved by adding 96% ethanol for 10 min, OD595 of the samples in a new plate were recorded (SpectraMax 190, San Jose, CA, USA). The percent of biofilm inhibition was calculated by comparing the optical density values for the treated samples and the untreated control [32].

### 3.9. RT-PCR

To verify the effect of semi-purified CFS of strain XN2 on the quorum sensing (QS) system of *S. aureus* CICC 10384, RT-PCR was developed for the detection of transcriptional changes observed in the *luxS* gene (encoding the synthase of QS signal molecular (Al-2) of *S. aureus*) and induced by 0 (control), 1/2 MIC or 2 MIC of semi-purified CFS of strain XN2. One loop of stain *S. aureus* CICC 10384 was added onto 10 mL sterilized LB broth and cultured at 30 °C for 12 h. The OD_600_ value was adjusted to 0.5 and inoculated into the fresh LB broth containing 0 μg/mL (control), 1/2 MIC, or 2 MIC semi-purified CFS of strain XN2 with the inoculum size of 3%, *v*/*v*. After co-culture in LB broth at 30 °C for 24 h, the gene expression of *luxS* was investigated. 

The total RNA from *S. aureus* CICC 10384 was extracted by the Trizol method (Cui et al., 2012) [33]. The reverse transcription reaction system (10 μL) contained: 2 μL of total RNA, 0.5 μL of dNTP, 0.5 μL of random primers, and 4 μL of distilled water without RNAase. All samples were heated at 70 °C for 5 min and then placed immediately in an ice bath. Then, 2 μL of 5× reverse transcription buffer, 0.5 μL of RNAase inhibitor, and 0.5 μL of MMLV reverse transcription enzyme were added immediately. The reverse transcription reaction procedure was as follows: 30 °C for 10 min, 42 °C for 1 h, 70 °C for 15 min, and 4 °C forever. The DNA templates were stored at −20 °C until use.

PCR reaction contained: 0.2 μL of upstream primer (10 μM), 0.2 μL of downstream primer (10 μM), 5 μL of 2× Ultra SYBR Mixture, 3.6 μL of double-distilled H_2_O, and 1 μL of 5× DNA templates. The PCR reaction conditions were: 95 °C for 10 min, 95 °C for 15 s, 60 °C for 1 min, and 40 cycles. Primers were designed according to the gene sequence of *luxS* Primer Premier 5.0 software (version 5.0, Primer Premier, Canada, 2017) as (5′-GAGATCTTATGCCATCAGTAGAAAG-3′; 5′-GGTCACCTTTATCCAAACACTTTCTC-3′).

### 3.10. Bacteriocin Purification

The semi-purified CFS of strain XN2 was additionally purified using an HPLC system with *photodiode array detector* (PDA) (UltiMate 3000, Dionex, Sunnyvale, CA, USA) and HC-C_18_ column (5 µm, 250 mm × 4.6 mm, Agilent Technologies, Palo Alto, CA, USA) [13] (Yue et al., 2013). The bacteriocin was eluted by linearly increasing the ACN content in ACN/water (Milli-Q) mixture from 10 to 95% over 40 min, at a flow rate of 0.5 mL/min with an injection volume of 1 mL. The amount of proteins was quantified according to the Bradford assay method [30]. The antibacterial activity was tested using an agar well diffusion assay, using *S. aureus* CICC10384 as an indicator strain [7]. The amino acids sequence were tested by N-sequence by Shengo bioengineering Co. Ltd. (Shanghai, China).

### 3.11. Statistical Analyses

SPSS 18.0 software (IBM SPSS Statistics, Amund, NY, USA) was used to analyze the data [16]. All data were the average representation of three measurements and shown as mean ± standard deviation. T-test was used to calculate whether there were significant differences in statistics for the group of data. t > t_0_ (*p* < 0.05) was considered that the differences (between the control and the testing groups, between testing groups) are significant.

## 4. Conclusions

In this study, one bacteriocin-producing strain, *Lacticaseibacillus rhamnosus* XN2, was successfully isolated from the yak yoghurt produced in the Xining and Qinghai Provinces in China. The bacteriocin, bacteriocin XN2, exhibited antibacterial activities against *Bacillus subtilis*, *B. cereus*, *Micrococcus luteus*, *Brochothrix thermosphacta*, *Clostridium butyricum*, *S. aureus*, *Listeria innocua CICC 10416*, *L. monocytogenes*, and *Escherichia coli*. The bacteriocin was able to disrupt the cell membrane (ultimately causing cell death), inhibit the secretion of α-haemolysin, and regulate the QS system of *S. aureus*. The amino acid sequence of the bacteriocin XN2 was YGNGVFSVIK.

## Figures and Tables

**Table 1:** Effect of enzymes, temperature, and pH on bacteriocin XN2.

	Testing Condition	Antibacterial Activities of Semi-Purified CFS of Stain XN2
	Lipase, α-Amylase,	+
	Proteinase K, Papain, α-Chymotrypsin, Trypsin, Pepsin	−
	Catalase	+
Effect of temperature and storage on the activity of semi-purified CFS of stain XN2	60 °C, 80 °C, and 100 °C	+
	37 °C for 14 d	+
	2 months at 4 °C	+
Effect of pH on the activity of semi-purified CFS of stain XN2	pH 2–8	+
	pH 9–10	−
The activity of semi-purified CFS of stain XN2 against gram-positive bacteria	*Bacillus subtilis* CICC 10034	+
	*B. cereus* CICC 2155	+
	*Micrococcus luteus* CICC 10209	+
	*Brochothrix thermosphacta* CICC 10509	+
	*Clostridium butyricum* CICC 10350	+
	*Staphylococcus aureus* CICC 10384	+
	*S. aureus* CICC 10201	+
	Methicillin-resistant S. aureus *	+
	*Listeria innocua* CICC 10416	+
	*L. monocytogenes* CICC 21529	+
The activity of semi-purified CFS of stain XN2 against gram-negative bacteria	*Escherichia coli* CICC 10302	+
	*E. coli* CGMCC 3373	+
	*E. coli* CICC 10300	+
	*Pseudomonas aeruginosa* CICC 21636	−
	*Enterobacter cloacae* CICC 21539	−
	*Salmonella paratyphi* β CICC 10437	−
The activity of semi-purified CFS of stain XN2 against fungi	*Aspergillus niger* CICC 2124	−
	*Candida albicans* CICC 1965	−
	*Saccharomyces cerevisiae* CICC 1002	−

CICC: China Center of Industrial Culture Collection. *: Methicillin-resistant *S. aureus* provided by a local hospital in Xi’an, Shaanxi, China. +: There is a clear inhibition circle. −: there is no inhibition circle.

## Data Availability

The data presented in this study are available on request from the corresponding author.

## Sample Availability

Samples of the compounds are available from the authors.
